# Experimental evolution reveals contrasting adaptive landscapes in lab and field environments

**DOI:** 10.1128/mbio.01700-26

**Published:** 2026-09-08

**Authors:** Alexander B. Chase, Zhao Wang, Claudia Weihe, Steven D. Allison, Jennifer B. H. Martiny

**Affiliations:** 1 Department of Earth Sciences, Southern Methodist University 2765, Dallas, Texas, USA; 2 Department of Ecology and Evolutionary Biology, University of California 189205, Irvine, California, USA; 3 Department of Earth System Sciences, University of California 8788 , Irvine, California, USA; Johns Hopkins University, Baltimore, Maryland, USA

**Keywords:** experimental evolution, microbial adaptation, field evolution, intergenic mutations, genetic parallelism, warming

## Abstract

Experimental evolution is widely used to infer microbial responses to environmental change, yet most laboratory studies impose constant, well-mixed conditions that differ fundamentally from fluctuating, spatially structured field environments. We compared genomic evolution in the leaf litter-associated bacterium *Curtobacterium* strain MMLR14_002 under control and warming treatments in laboratory culture and in a complementary field experiment. Laboratory-derived isolates accumulated more mutations per genome and exhibited stronger locus-level parallelism, with mutations recurring in a small number of coding loci. Field-derived isolates accumulated fewer mutations per genome, and these mutations rarely occurred in the same coding loci across replicate populations. Instead, field isolates exhibited a higher proportion of intergenic mutations, with mutations recurring in the same intergenic regions across independent field deployments. When coding mutations were detected in the field, they were distributed across functionally diffuse targets and more often involved metabolic pathways than the core cellular processes repeatedly targeted during laboratory evolution. Warming itself did not consistently influence mutation accumulation or the genomic distribution of mutations; instead, laboratory and field contexts primarily shaped the accumulation, targets, and repeatability of genomic change. These results suggest that laboratory thermal evolution identifies adaptive routes favored under sustained selection but may overestimate coding-level parallelism under heterogeneous field conditions. Bridging laboratory and field evolution will likely require experimental designs that incorporate temporal variability and spatial heterogeneity characteristic of natural systems.

**IMPORTANCE:**

A central goal of experimental evolution is to infer how microbes evolve in nature from laboratory studies. Here, we evaluate this assumption by comparing genomic evolution of a leaf litter-associated *Curtobacterium* strain in laboratory and field warming experiments to identify broad patterns rather than isolate the contribution of any single environmental factor. We find that the strong parallelism at coding loci observed under laboratory conditions is reduced in the field, while mutations recurring in the same intergenic regions across field deployments suggest that parallel evolution in nature may more often involve regulatory noncoding regions rather than coding targets. These results show that environmental context reshapes adaptive landscapes and may limit the parallelism of coding-level genomic responses inferred from homogeneous laboratory conditions.

## OBSERVATION

Experimental evolution is widely used to infer how microbial populations respond to environmental change (1, 2). Many laboratory experiments are conducted in well-mixed media under constant temperatures that promote growth and sustained directional selection (3, 4). Under these conditions, repeated mutations in specific loci associated with core growth and regulatory functions produce strong genetic parallelism across replicate populations (5).

These conditions differ from natural environments. In soil, microbes inhabit spatially structured substrates where closely related bacterial lineages can remain genetically differentiated across microhabitats due to limited gene flow (6). Temperature also covaries with moisture and resource availability through time (7, 8). Together, this spatial and temporal variation generates heterogeneous selective pressures, suggesting that field environments may reshape adaptive landscapes and alter evolution relative to constant laboratory conditions (9, 10).

**Fig 1:**
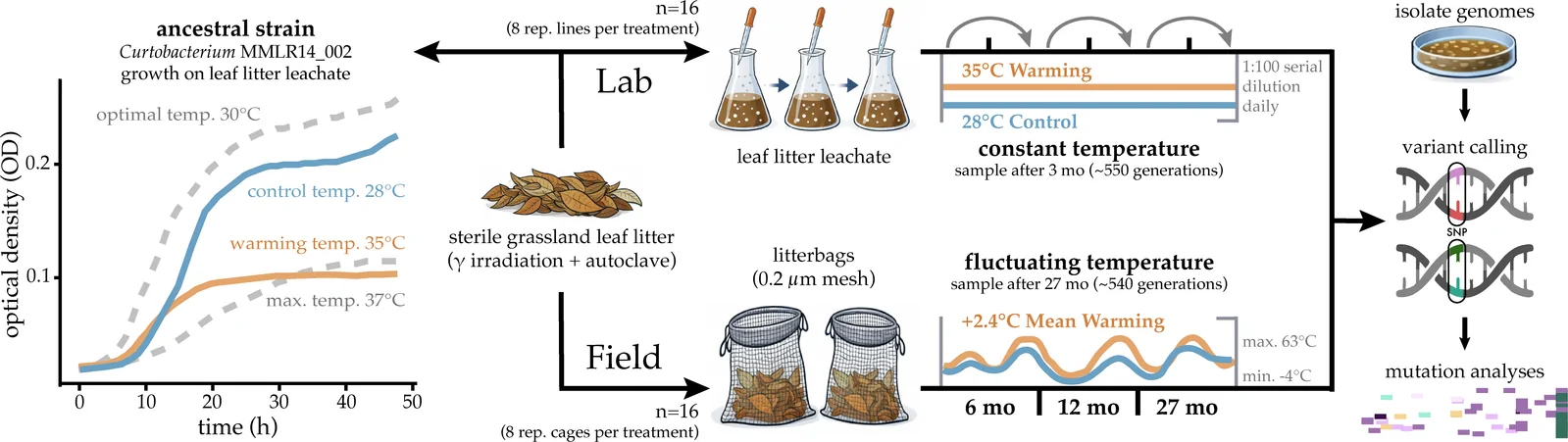
Experimental design contrasting laboratory and field warming. The same focal bacterial strain, *Curtobacterium* MMLR14_002, was evolved under laboratory and field conditions using the same grassland leaf litter-derived substrate. Laboratory populations were propagated in leaf litter leachate under constant control and warming temperatures (28°C control; 35°C warming) for an estimated 548 generations. The 28°C control was near the ancestral strain’s optimal growth temperature (~30°C), while 35°C approached its upper growth limit (~37°C). Field populations were inoculated into sterile grassland leaf litter enclosed in microbial cages and exposed to ambient or experimentally warmed conditions (+2.4°C mean warming; +5.3°C maximum warming) within a spatially structured litter matrix over 27 months, corresponding to an estimated 538 cumulative generations. Evolved lineages were re-isolated through time and sequenced to compare mutation accumulation, genomic distribution of mutations, and genetic parallelism across environments.

To evaluate how environmental context influences evolutionary outcomes, we compared genomic evolution of a soil bacterium under control and warming treatments in laboratory and field settings (Fig. 1). *Curtobacterium* strain MMLR14_002 was isolated from grassland leaf litter at the Loma Ridge Global Change Experiment (LRGCE; CA, USA; 33.737°N, 117.695°W), where *Curtobacterium* is among the most abundant bacterial genera (11) and has been used previously to study bacterial responses to environmental change (12, 13). Field populations were inoculated into sterile grassland leaf litter enclosed in microbial “cages” (14) and deployed for 27 months under ambient or experimentally warmed field conditions at LRGCE. Field warming was achieved using small greenhouse-style enclosures (Δ*T*_mean_ = +2.4°C; Δ*T*_max_ = +5.3°C), while retaining temporal variation in temperature and moisture within a spatially structured litter matrix (Fig. S1). Laboratory populations were propagated in well-mixed leachate from the same grassland litter substrate under constant temperatures (control = 28°C, warmed = 35°C).

By re-isolating evolved lineages through time and sequencing individual genomes, we compared mutation accumulation, genomic distribution of mutations, and genetic parallelism across laboratory and field environments. This comparison contrasts constant, well-mixed laboratory conditions with a field deployment in which warming occurred within a spatially structured and temporally variable litter environment.

## RESULTS AND DISCUSSION

**Fig 2:**
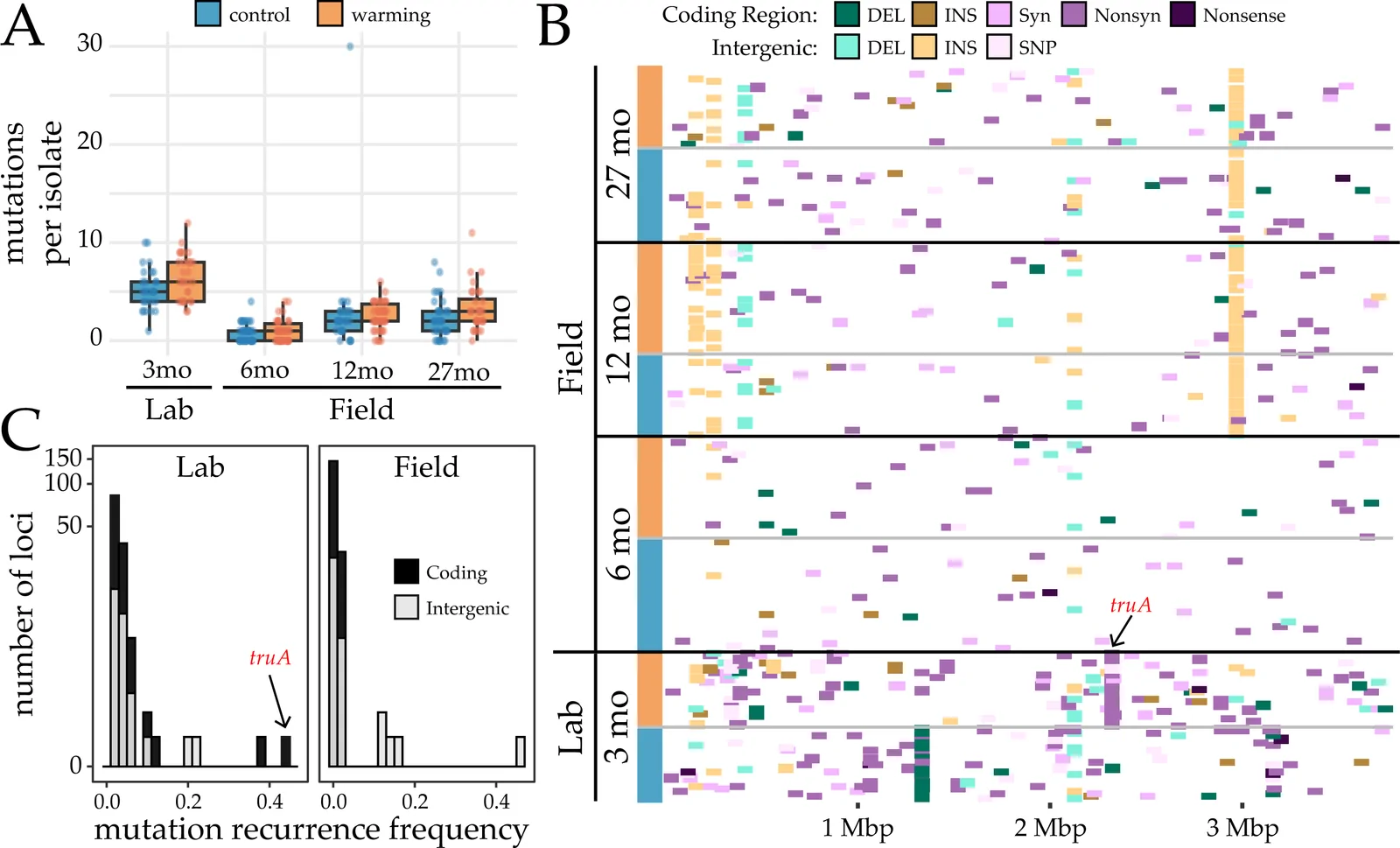
Contrasting evolutionary dynamics in laboratory and field environments. (**A**) Number of mutations per isolate across experimental contexts. Laboratory-derived isolates accumulated significantly more mutations than field-derived isolates, while sequenced field isolates accumulated mutations gradually over the 27-month deployment. (**B**) Genome-wide map of mutation positions across isolates. Laboratory populations exhibit strong locus-level parallelism in coding regions, including treatment-specific repeated mutation of coding loci (e.g., *truA* in laboratory warming, shown in red). (**C**) Distribution of mutation recurrence frequencies across loci. The *x*-axis shows the proportion of isolates with a mutation at a given locus, and the *y*-axis shows the number of loci (note log scale). Bars indicate coding (black) and intergenic (gray) regions, with laboratory and field experiments shown separately. Experimental contexts are pooled across treatments and field time points; therefore, treatment-specific laboratory recurrence is diluted in this panel. For example, *truA* was mutated in 87.1% of laboratory warming isolates (Fig. S4), but lower recurrence when laboratory treatments were pooled.

Laboratory-derived lineages accumulated more mutations per genome than field-derived lineages (Fig. 2A; mean ± SD: lab = 5.90 ± 2.3, field_ALL_ = 1.99 ± 2.5, field_T3_ = 2.69 ± 2.1; negative binomial generalized linear model, *P* < 0.001). After accounting for estimated cumulative generations, laboratory isolates accumulated 1.44 times as many mutations as field isolates (*P* < 0.01). The laboratory experiment and final field sampling point represented broadly similar estimated cumulative generations (548 vs 538), despite large differences in elapsed time (83 vs 808 days). Field generation estimates were based on independent litterbag sampling and likely represent an upper bound for cumulative growth (see Supplemental Methods). Within the field experiment, isolates accumulated mutations over time (Fig. 2A; *P* < 0.001), with mutation counts increasing by an estimated 17.9% per 100 cumulative generations and approximately twofold over the 27-month deployment.

In contrast to the clear difference between the laboratory and field, warming did not strongly alter mutation accumulation. Warming was associated with slightly higher mutation counts in a simple additive model including both laboratory and field isolates (count ratio = 1.2; *P* < 0.05), but this effect was not significant in models that allowed the warming effect to differ between laboratory and field contexts or accounted for estimated cumulative generations (all *P* > 0.1). Flow cytometry also indicated that total cell abundances did not differ between ambient and warmed field treatments at sampled time points (Fig. S2), although abundances declined over time across both field treatments (paired *t*-test, *P* < 0.001). Thus, differences in field mutation accumulation were not explained by treatment-specific differences in total abundance. Greater mutation accumulation in laboratory isolates may instead reflect more frequent population bottlenecks imposed by daily transfer and more homogeneous selective conditions compared with the field environment (13, 15).

Laboratory and field populations also differed in the genomic distribution and functional composition of mutations. Compared with field isolates, laboratory isolates were 27-fold more likely to harbor nonsynonymous or nonsense mutations in protein-coding genes (Fig. S3A; likelihood ratio test, *P* < 0.001), consistent with higher nonsynonymous-to-synonymous mutation ratios (N/S_LAB_ = 2.9–4.0; N/S_FIELD_ = 1.3–3.2). Conversely, field isolates accumulated a higher proportion of intergenic mutations (Fig. S3B; field = 49.6% ± 39.1%, lab = 24.3% ± 18.3%; Wilcoxon rank‐sum, *P* < 0.001). In laboratory populations, coding mutations disproportionately affected genes involved in genetic information processing and core cellular processes, consistent with modification of core growth-related functions under constant conditions (16). Coding mutations in field populations were enriched in metabolic genes, consistent with adaptation to variable resource environments (17).

Because parallel evolution across independent populations often reflects positive selection (18), we identified mutations that recurred across treatments and environmental contexts (Fig. 2B; Table S1). Laboratory isolates showed greater overlap in mutated coding loci than field isolates, with mean Jaccard similarity of 0.12–0.13 across laboratory treatments compared with 0–0.02 across field treatments. Repeated targeting of coding loci was also most pronounced within laboratory treatments: the most frequently mutated coding locus occurred in 73.3% of laboratory control isolates and 87.1% of laboratory warming isolates, whereas no coding locus was mutated in more than 4.1% of isolates in either field treatment. Under laboratory warming, the most frequently targeted coding locus was *truA* (tRNA pseudouridine synthase A), while under ambient laboratory conditions, it was a locus encoding a hypothetical protein; neither locus was mutated in field isolates (Fig. S4). Mutations recurring in field isolates occurred primarily in intergenic regions (Fig. 2B and C), indicating parallelism at noncoding targets, although no field region was mutated in a proportion of isolates comparable to the most frequently targeted laboratory coding loci (Table S1). These recurrent intergenic mutations are consistent with the adaptive role of regulatory evolution observed across diverse systems (19, 20).

To determine whether the field-evolved targets were reproducible, we further compared our results with an independent field evolution experiment in which the same ancestral *Curtobacterium* strain was transplanted across a climatic gradient (13) that included the grassland site studied here. Despite the same starting genotype, none of the coding loci repeatedly mutated here (Table S1) overlapped with those reported in the gradient experiment, including mutations detected from re-isolated clones or inferred from population-level read mapping. In contrast, several intergenic regions were repeatedly mutated across studies (Table S2). Some of these shared regions reached high isolate frequencies across independent deployments, including three of the most commonly mutated regions identified here. At least one intergenic region also rose to high population allele frequencies at specific climate gradient sites. These patterns suggest that intergenic regions may be more consistent targets of parallel evolution in the field than coding regions. However, the frequencies of these shared intergenic mutations varied across sites and measurement scales, consistent with spatial structure, limited mixing, and local environmental history constraining the spread of beneficial alleles.

## CONCLUSION

Laboratory evolution under constant, well-mixed conditions produced stronger parallelism at a few coding loci, whereas field evolution involved a broader distribution of genomic targets, including recurrent intergenic mutations that were shared across independent field studies but varied in frequency across sites and populations. While we cannot identify the specific drivers of these different outcomes between the laboratory and field experiments, the patterns suggest that spatial and temporal variation alters the adaptive landscape by reducing repeated mutation of the same coding loci favored under homogeneous laboratory conditions. Thus, laboratory evolution may overestimate coding-level parallelism under heterogeneous field conditions (21). Future laboratory experiments that incorporate temporal variability and spatial heterogeneity may improve inference about microbial evolution in natural field systems (22).

## Data Availability

The reference genome for *Curtobacterium* strain MMLR14_002 is available under the National Center for Biotechnology Information BioProject PRJNA524191. All other data and relevant code used can be found at GitHub, https://github.com/alex-b-chase/evol-fieldVlab.

## SUPPLEMENTAL MATERIAL

Supplemental Material mbio.01700-26-s0001.pdf Supplemental methods, figures, and table captions.

Table S1 mbio.01700-26-s0002.xlsx Most frequently mutated loci across laboratory and field experimental contexts.

Table S2 mbio.01700-26-s0003.xlsx Cross-study comparison of recurrent intergenic mutations.

ASM does not own the copyrights to Supplemental Material that may be linked to, or accessed through, an article. The authors have granted ASM a non-exclusive, world-wide license to publish the Supplemental Material files. Please contact the corresponding author directly for reuse.

## References

[1] Elena SF, Lenski RE. 2003. Evolution experiments with microorganisms: the dynamics and genetic bases of adaptation. Nat Rev Genet 4:457–469. doi:10.1038/nrg1088 12776215

[2] Barrick JE, Lenski RE. 2013. Genome dynamics during experimental evolution. Nat Rev Genet 14:827–839. doi:10.1038/nrg3564 PMC4239992 24166031

[3] Tenaillon O, Rodríguez-Verdugo A, Gaut RL, McDonald P, Bennett AF, Long AD, Gaut BS. 2012. The molecular diversity of adaptive convergence. Science 335:457–461. doi:10.1126/science.1212986 22282810

[4] Bennett AF, Dao KM, Lenski RE. 1990. Rapid evolution in response to high-temperature selection. Nature 346:79–81. doi:10.1038/346079a0 2195353

[5] Kawecki TJ, Lenski RE, Ebert D, Hollis B, Olivieri I, Whitlock MC. 2012. Experimental evolution. Trends Ecol Evol 27:547–560. doi:10.1016/j.tree.2012.06.001 22819306

[6] Chase AB, Arevalo P, Brodie EL, Polz MF, Karaoz U, Martiny JBH. 2019. Maintenance of sympatric and allopatric populations in free-living terrestrial bacteria. mBio 10:e02361-19. doi:10.1128/mBio.02361-19 PMC6819660 31662456

[7] Sant DG, Smith TP, Wong ELY, Cohen J, King KC, Bell T, Barraclough TG. 2025. Eco-evolutionary robustness of wild bacterial communities to experimental perturbation. ISME J 19:wraf144. doi:10.1093/ismejo/wraf144 PMC12743297 40693750

[8] Martiny JBH, Martiny AC, Brodie E, Chase AB, Rodríguez-Verdugo A, Treseder KK, Allison SD. 2023. Investigating the eco-evolutionary response of microbiomes to environmental change. Ecol Lett 26:S81–S90. doi:10.1111/ele.14209 36965002

[9] Kaminsky LM, Burghardt L, Bell TH. 2025. Evolving a plant-beneficial bacterium in soil vs. nutrient-rich liquid culture has contrasting effects on in-soil fitness. Appl Environ Microbiol 91:e02085-24. doi:10.1128/aem.02085-24 PMC12016532 40067020

[10] Montbel V, Hrcek J. 2026. Experimental evolution in communities: beyond pairwise interactions. Evolution 80:889–901. doi:10.1093/evolut/qpag029 41746203

[11] Matulich KL, Weihe C, Allison SD, Amend AS, Berlemont R, Goulden ML, Kimball S, Martiny AC, Martiny JBH. 2015. Temporal variation overshadows the response of leaf litter microbial communities to simulated global change. ISME J 9:2477–2489. doi:10.1038/ismej.2015.58 PMC4611511 25978544

[12] Chase AB, Karaoz U, Brodie EL, Gomez-Lunar Z, Martiny AC, Martiny JBH. 2017. Microdiversity of an abundant terrestrial bacterium encompasses extensive variation in ecologically relevant traits. mBio 8:e01809-17. doi:10.1128/mBio.01809-17 PMC5686540 29138307

[13] Chase AB, Weihe C, Martiny JBH. 2021. Adaptive differentiation and rapid evolution of a soil bacterium along a climate gradient. Proc Natl Acad Sci USA 118:e2101254118. doi:10.1073/pnas.2101254118 PMC8106337 33906949

[14] Allison SD, Lu Y, Weihe C, Goulden ML, Martiny AC, Treseder KK, Martiny JBH. 2013. Microbial abundance and composition influence litter decomposition response to environmental change. Ecology 94:714–725. doi:10.1890/12-1243.1 23687897

[15] Kaminsky LM, Sadeghi J, Grandinette E, Bell TH. 2026. The adaptation chip: repurposing the principles of the ichip for guiding *in situ* experimental evolution. ISME Commun 6:ycag053. doi:10.1093/ismeco/ycag053 PMC13064666 41971532

[16] Kvitek DJ, Sherlock G. 2013. Whole genome, whole population sequencing reveals that loss of signaling networks is the major adaptive strategy in a constant environment. PLoS Genet 9:e1003972. doi:10.1371/journal.pgen.1003972 PMC3836717 24278038

[17] Hesse E, Luján AM, O’Brien S, Newbury A, McAvoy T, Soria Pascual J, Bayer F, Hodgson DJ, Buckling A. 2024. Parallel ecological and evolutionary responses to selection in a natural bacterial community. Proc Natl Acad Sci USA 121:e2403577121. doi:10.1073/pnas.2403577121 PMC11388356 39190353

[18] Christin P-A, Weinreich DM, Besnard G. 2010. Causes and evolutionary significance of genetic convergence. Trends Genet 26:400–405. doi:10.1016/j.tig.2010.06.005 20685006

[19] Stern DL, Orgogozo V. 2008. The loci of evolution: how predictable is genetic evolution? Evolution 62:2155–2177. doi:10.1111/j.1558-5646.2008.00450.x PMC2613234 18616572

[20] Wray GA. 2007. The evolutionary significance of *cis*-regulatory mutations. Nat Rev Genet 8:206–216. doi:10.1038/nrg2063 17304246

[21] Kassen R. 2024. Experimental evolution and the nature of biodiversity. Oxford University Press.

[22] Simpson AMR, Chase AB, Rodríguez-Verdugo A, Martiny JBH. 2025. Investigating bacterial evolution in nature with metagenomics. Curr Opin Microbiol 87:102654. doi:10.1016/j.mib.2025.102654 40811873

