# Supplementary material for: Experimental evolution reveals contrasting adaptive landscapes in lab and field environments
Source: mBio. 2026 Sep 8;17(10):e01700-26. doi: 10.1128/mbio.01700-26 (PMC13712956; doi:10.1128/mbio.01700-26)
Supplement: Supplementary file 1 [file mbio.01700-26-s0001.pdf]

**Supplementary Information for**

# Experimental evolution reveals contrasting adaptive landscapes in lab and field environments

Alexander B. Chase[1]*, Zhao Wang[2], Claudia Weihe[2], Steven D. Allison[2,3], Jennifer B. H. Martiny[2]

[1]Department of Earth Sciences, Southern Methodist University, Dallas, TX, USA
[2]Department of Ecology and Evolutionary Biology, University of California, Irvine, CA, USA
[3]Department of Earth System Sciences, University of California, Irvine, CA, USA

*Corresponding author: Alexander B. Chase: abchase@smu.edu

## Materials and Methods

### Experimental design

A single focal bacterial strain, *Curtobacterium* MMLR14_002, was used in both laboratory and field evolution experiments to enable direct comparison of genomic evolutionary dynamics across experimental contexts. *Curtobacterium* is a cosmopolitan soil bacterium (1) and one of the most abundant genera at the grassland field site, the Loma Ridge Global Change Experiment (LRGCE) in southern California (2). In prior studies, we have extensively characterized *Curtobacterium* MMLR14_002, and *Curtobacterium* more broadly, for their physiological and genomic potential to respond to environmental change, including nitrogen amendments and drought (3–5). *Curtobacterium* MMLR14_002 was originally isolated from grassland leaf litter at the study site in 2011, and its complete reference genome has been reported previously (NCBI BioProject PRJNA524191) (1, 5).

To parameterize the laboratory evolution experiment, we measured growth of the ancestral strain in leaf litter medium across a temperature gradient (24, 28, 30, 32, 35, and 37°C) over a 3-day period; no growth was detected at 40°C. Maximum culture yield occurred at 30°C, whereas maximum growth rate occurred at 28°C, which was therefore used as the laboratory control temperature. Growth curves also showed that exponential growth ended after approximately 35 h at 28°C and 40 h at 35°C, with growth at 35°C depressed relative to lower temperatures and consistent with incubation near the strain's thermal maximum (Fig. 1). These growth rate estimates, together with the dilution ratio used in the evolution experiment, were used to determine the transfer interval and estimate that laboratory populations experienced approximately 6.6 generations per day.

To provide context for *in situ* growth dynamics, we also considered independent field growth-rate estimates from a short-term litterbag experiment conducted with two litter-derived *Curtobacterium* strains sharing >95% ANI with the focal strain, including strain MCLR17_058 isolated from the LRGCE (Cabrera et al., unpublished data). In that experiment, strains MCBD17_026 and MCLR17_058 were inoculated into field litterbags and tracked by flow cytometry every 2-4 days over a 2-month period. Estimated growth rates were 0.675 ± 0.0555 and 0.655 ± 0.0473 generations per day, respectively. Although these estimates were not obtained for the focal strain itself, they provide an approximate benchmark indicating that *in situ* growth in litterbags is substantially slower than growth under laboratory conditions.

Laboratory and field experiments were conducted independently but followed parallel replicate designs, with evolution tracked through isolate-based whole-genome sequencing. For both experiments, the same batch of grassland leaf litter was collected outside the experimental plots at LRGCE. *Curtobacterium* MMLR14_002 was streaked from a glycerol stock onto grassland litter medium, and a single colony was used to inoculate 3 mL of litter medium. After two days, the ancestral strain was subsampled for resequencing, and the remaining culture was partitioned for the field and laboratory experiments. For the field experiment, the starting culture was washed three times with 0.9% saline solution to remove residual medium before inoculation. The laboratory inoculum was not washed because the same medium was used. Cultures were normalized by optical density ($OD_{600}$) across both experimental designs prior to inoculation.

Laboratory evolution experiment. Laboratory evolution was conducted under constant temperature conditions using leaf litter leachate medium to facilitate comparison with field observations from leaf litter microbial cages (see below). Eight independent replicate populations were propagated daily under control (28°C) and warming (35°C; near the *Curtobacterium* thermal maximum, Fig. 1) treatments for approximately three months (83 days), corresponding to ~547.8 generations based on doubling-time estimates from growth curves in litter leachate (see above). Populations were maintained in 3 mL of litter medium under standard laboratory conditions (constant shaking at 150 rpm), with daily transfers to fresh medium at a 1:100 dilution to sustain exponential growth. At the end of the experiment, four isolates were randomly sampled from each replicate population, resulting in 64 laboratory-evolved isolates in total (8 replicates x 2 treatments x 4 isolates). Isolates were grown in LB medium, pelleted, and stored at -20°C in glycerol until DNA extraction.

Field evolution experiment. Field evolution was conducted using microbial cages deployed in the grassland field site at LRGCE in December 2020. Cages consisted of 6 g of sterilized grassland leaf litter (gamma irradiated) inoculated with 1.5 mL of washed cell suspension of *Curtobacterium* MMLR14_002 (see above) and enclosed within 0.2 $\mu$m mesh bags, which prevent microbial dispersal and immigration while allowing exchange of water and dissolved solutes with the surrounding environment (6). Eight independent replicate cages were established under ambient conditions and eight under experimental warming. Warming was achieved using small greenhouse-style enclosures that increased mean temperature by approximately 2.4°C relative to ambient conditions, with a maximum temperature difference of +5.3°C (Fig. 1; Fig. S1). Field cages were deployed until March 2023 (808 days) and sampled at three time points (6, 12, and 27 months), corresponding to up to ~537.6 estimated generations based on independent field growth-rate estimates in litterbags (unpublished data from Cabrera et al.). At each time point, 3 g of leaf litter were collected and transported back to the laboratory for re-isolation (0.1 g) and flow cytometry (0.1 g) in sterile Whirl-Pak bags kept on ice until storage at -20°C. The remaining 3 g were transferred into new microbial cages containing 3 g of sterilized leaf litter, resealed, and redeployed at LRGCE. For re-isolation, 0.1 g of collected litter was resuspended in 1.5 mL of 0.9% saline solution, vortexed for 1 min, serially diluted ($10^{-2}$ and $10^{-4}$), and plated on LB agar. For each microbial cage and treatment, six isolates were picked, pelleted, and stored in glycerol until further processing. In total, we recovered 288 field isolates (8 replicates x 2 treatments x 6 isolates x 3 time points).

To assess whether differences in mutation accumulation could be explained by population collapse or differential mortality, total cell abundances were quantified using flow cytometry, as previously described (7). Briefly, 0.1 g of collected litter were suspended in 5 mL of 1% GTA solution and sonicated for 30 min prior to filtration through a 2.7 $\mu$m glass fiber filter. Filtered cells were fixed, stained with standard nucleic acid dyes, and analyzed by flow cytometry. Culturable abundances were additionally estimated from colony forming units (CFUs) at 6 months to validate flow cytometry measurements (Fig. S2).

Temperature within field litterbags was continuously monitored using iButton temperature sensors placed inside representative cages. Temperature data were recorded at 5-min intervals throughout the deployment and used to verify sustained warming relative to ambient controls (Fig. S1).

**DNA extraction and genome analyses**

Genomic DNA was extracted from frozen pellets from both the laboratory and field isolates using the Wizard Genomic DNA Purification Kit (Promega; Madison, WI) with the additional step of adding lysozyme for Gram-positive bacteria. Extracted DNA was quantified on the Qubit (BioTek; Winooski, VT) and quality assessed on the Nanodrop (Thermo Fisher; Waltham, MA) prior to library preparation. Whole-genome sequencing libraries were prepared using the Nextera XT Library Preparation kit (Illumina Inc., San Diego, CA), pooled in equimolar portions, and sequenced on an Illumina NovaSeq 6000 platform to generate 150 bp paired-end reads. Based on raw read output, the average expected coverage was approximately 328x per sample, which was sufficient for confident identification of single-nucleotide polymorphisms (SNPs) and small insertions/deletions (INDELs) relative to the ancestral reference genome. To reduce false positives arising from alignment artifacts or reference-specific issues, we re-sequenced the ancestral strain (*Curtobacterium* MMLR14_002) from the inoculum across four independent sequencing runs and identified problematic sites (e.g., sequencing artifacts) that were masked using reference-specific exclusion files.

<u>Read processing and quality filtering</u>. Paired-end Illumina reads were quality filtered and trimmed using a standardized preprocessing pipeline with BBMap v38.96 (8). Reads failing minimum quality or length thresholds were removed prior to assembly and mapping. Only samples with complete paired read files and sufficient read depth were retained for downstream analyses, resulting in an average expected coverage of 205x per sample. To initially verify the recovery of the ancestral strain, *de novo* assemblies with SPAdes v4.0.0 (9) were generated from quality-filtered reads to assess genome completeness and detect potential contamination. Assemblies were compared against a curated reference genome set (10, 11) using FastANI v1.33 (12). Each isolate was assigned to the closest reference genome based on maximum ANI and only isolates with ≥99.9% ANI to the MMLR14_002 reference genome were retained for variant calling. Isolates failing quality control metrics, including abnormal GC content (<65 GC%), low coverage (<10 average depth) and/or incomplete assemblies, or evidence of contamination, defined as mixed taxonomic assignment based on taxon-annotated-GC-coverage (TAGC) plots assigned against the NCBI nucleotide database, were excluded from downstream analyses. After filtering, 301 isolates were retained for analysis.

<u>Variant Calling</u>. For each isolate, quality-filtered reads were mapped to the assigned reference genome using BWA-MEM v0.7.17 (13), with read group information specified per isolate. Alignments were sorted and indexed using samtools v1.20 (14), and duplicate reads were marked using Picard MarkDuplicates for consistency across samples, although duplicate rates were generally low for clonal isolates. Final BAM files were indexed and used for variant calling.

Variants were called independently for each isolate using GATK v 4.5.0.0 HaplotypeCaller with haploid ploidy specified (15). SNPs and indels were separated and subjected to hard filtering using standard GATK quality metrics (QD, FS, MQ, MQRankSum, ReadPosRankSum), followed by additional empirical filters on minimum depth (DP ≥ 10) and variant quality (QUAL ≥ 50). Variants failing any filter were excluded, along with variant calls overlapping with problematic sites from the re-sequenced ancestral strain (see above). To further validate variant calls, all isolates were independently analyzed using breseq v0.38.3 under default isolate-mode settings

(16) and cross-compared with GATK to ensure concordance. Discrepant calls were inspected manually, and downstream analyses were restricted to high-confidence mutations supported by consistent evidence across methods.

Filtered VCFs were annotated using gdtools ANNOTATE against the reference genome, with protein coding regions and annotations assigned with Prokka v1.13 (17). Because gdtools annotation outputs can vary slightly in column structure across mutation types, a custom Python script was used to extract and standardize relevant fields across samples, including mutation type, genomic position, mutated gene(s), functional annotation, and quality metrics. All annotated mutations were concatenated into a master mutation table, with each mutation associated with a unique isolate identifier. Isolates with zero detected mutations were retained in downstream analyses to avoid biasing summary statistics.

Mutation and functional classification. Mutations were classified into functional categories based on annotation output. SNPs were classified as synonymous, nonsynonymous, nonsense, or intergenic. Indels were classified as insertions or deletions and further distinguished as coding or intergenic based on overlap with annotated genes. For analyses of parallelism and functional targeting, multiple mutations affecting the same gene within a single isolate were normalized to a single gene-level event.

Genes harboring coding mutations were functionally annotated using KEGG GhostKOALA (18), assigning KEGG Orthology (KO) identifiers where available. KO assignments were mapped to higher-level functional categories using the KEGG hierarchy. For visualization and statistical analyses, KO terms were collapsed into broad functional classes (e.g., metabolism; genetic information processing; cellular processes). Intergenic mutations were treated separately and not assigned functional categories.

**Statistical analyses**

All statistical analyses were conducted in R v4.2.2. Mutation counts per isolate were analyzed using negative binomial generalized linear models to account for overdispersion, with the effects of experimental context (laboratory vs. field), temperature treatment (warming vs. control), and time evaluated using model comparisons and likelihood-ratio tests. Differences in total cell abundance between warming and control treatments were assessed separately for each count type (flow cytometry and colony-forming units) and time point using t-tests with Benjamini-Hochberg correction.

Differences in the proportion of isolates harboring coding mutations were tested using binomial generalized linear models, while enrichment of nonsynonymous relative to synonymous mutations and treatment-specific enrichment of recurrently mutated genomic regions were evaluated using Fisher’s exact tests. Differences in the proportion of intergenic mutations per isolate were assessed using Wilcoxon rank-sum tests.

## Supplemental Figures and Tables

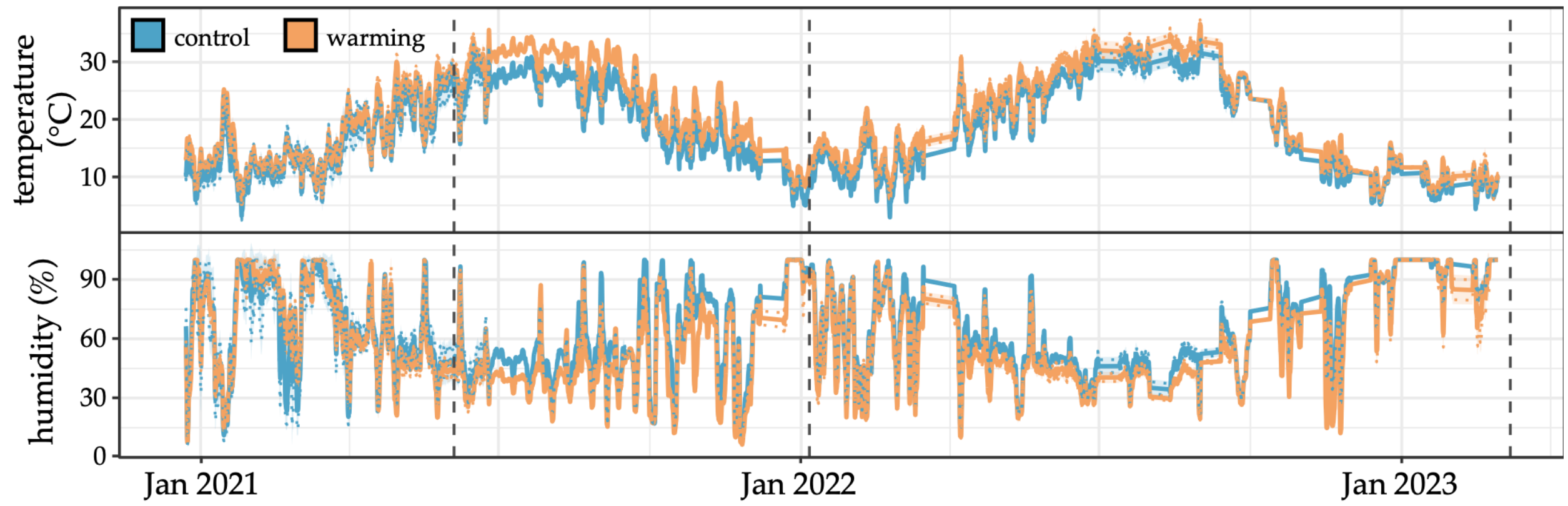


**Figure S1**. **Microclimatic conditions within field litterbags during the 27-month deployment.** Temperature and relative humidity were measured using iButton sensors placed inside litterbags. Time series show smoothed treatment-level means for control and warming plots (n = 8 plots per treatment). Shaded ribbons indicate ±1 standard deviation among plots, and dotted lines denote smoothed minimum and maximum values across plots at each time point. Vertical dashed lines indicate litterbag sampling at approximately 6, 12, and 27 months. Warming plots were consistently warmer and drier than controls, with an average paired increase of 2.35 ± 1.21°C and a decrease of 5.09 ± 6.96% in relative humidity.

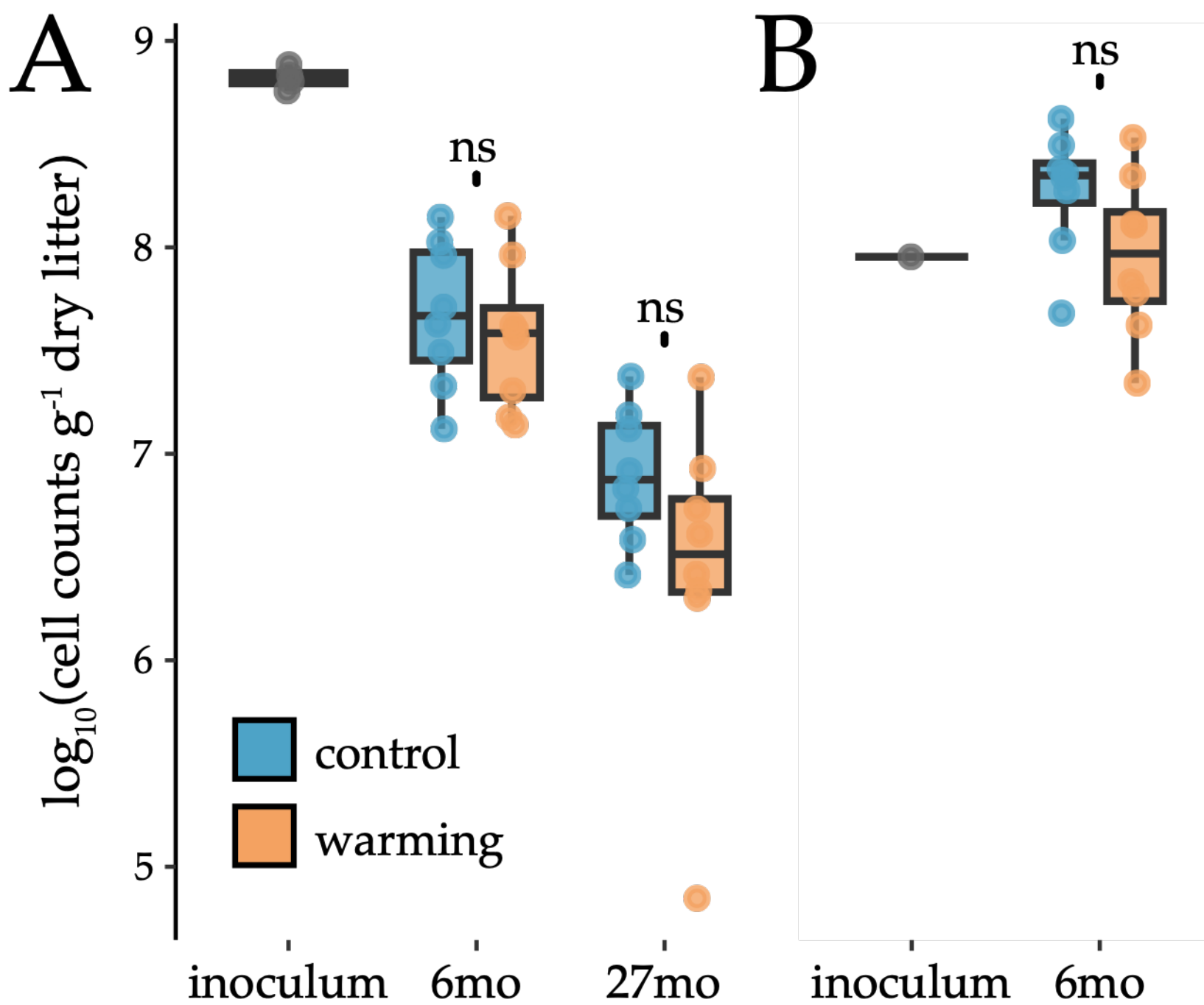


**Figure S2. Total cell abundance across field treatments**. **A)** Flow cytometry- and **B)** plate-based estimates of total cell abundance across treatments. Flow cytometry measurements were obtained for the inoculum and after 6 and 27 months of field incubation, whereas plate counts were obtained only after 6 months. Cell abundances did not differ significantly between warming and control treatments at any sampled time point for either count type, although flow cytometry counts declined over time, indicating that treatment effects on mutation accumulation were not driven by treatment-dependent differences in population size.

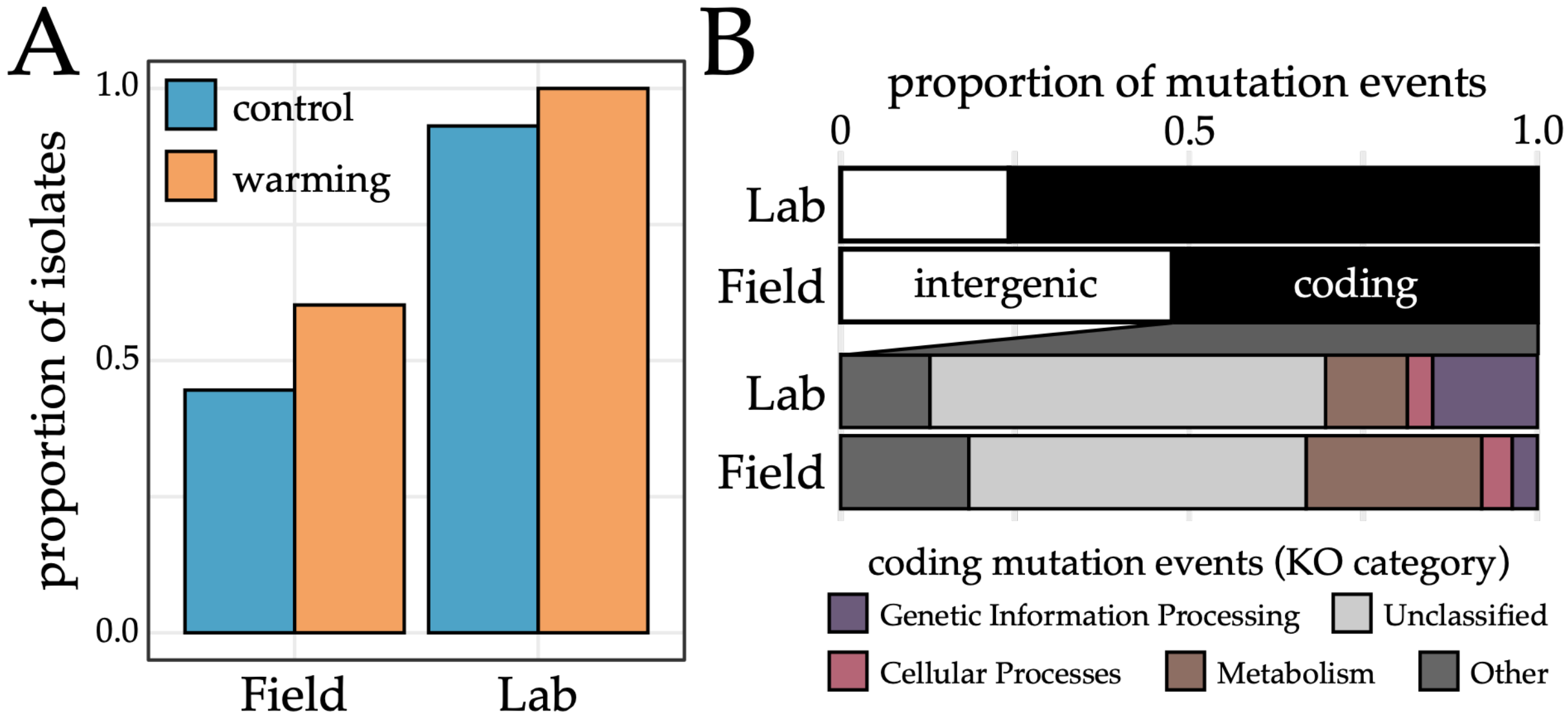


**Figure S3**. **Coding mutation prevalence and functional distribution**. **A)** Proportion of isolates containing at least one nonsynonymous or nonsense mutation in a protein-coding gene. Laboratory isolates were substantially more likely than field isolates to harbor coding mutations regardless of temperature treatment. **B)** Distribution of mutation events across coding and intergenic regions and functional gene categories. Bars show the proportion of mutation events occurring in coding versus intergenic regions, with coding mutations further partitioned by broad functional gene categories.

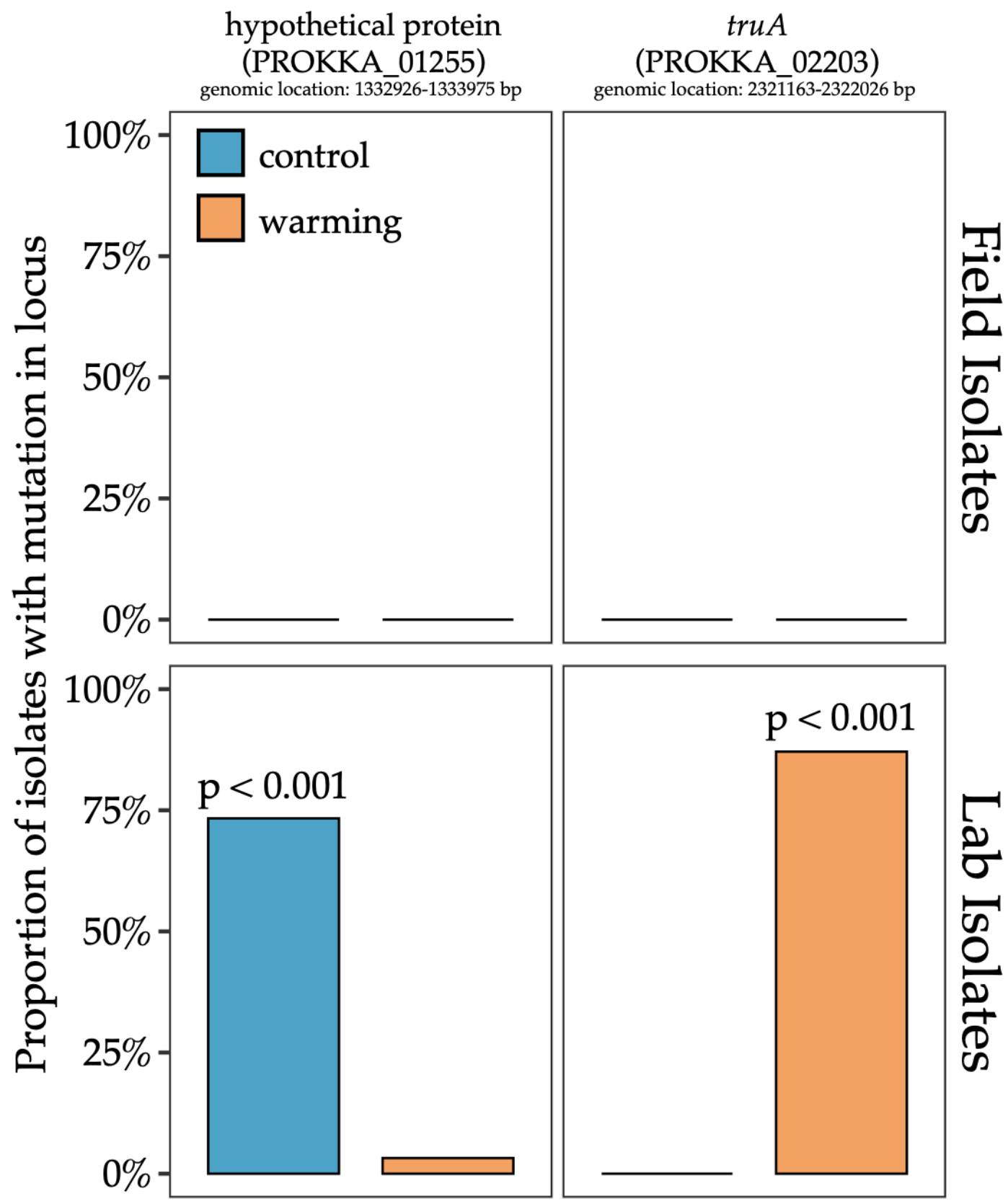


**Figure S4**. **Loci exhibiting strong parallelism in laboratory evolution experiments**. Genomic regions repeatedly mutated across independent laboratory isolates under warming or control conditions (Fig. 2B). These include parallel nonsynonymous mutations under laboratory warming in *truA* and repeated deletions or nonsynonymous mutations in a hypothetical protein under laboratory control. Neither locus was detected in field isolates.

**Table S1**. **Most frequently mutated loci across experimental contexts**. Summary of the ten most frequently mutated loci or intergenic regions across all isolates. For each region, the table reports gene annotation (when available), mutation class, and the number of independent isolates harboring at least one mutation, stratified by experimental context (laboratory control, laboratory warming, and field treatments across time points).

**Table S2**. **Cross-study comparison of recurrent intergenic mutations**. Recurrent intergenic insertion-deletion (INDEL) regions identified in this study were compared with mutations detected in a prior climate-gradient field experiment using the same ancestral *Curtobacterium* strain, MMLR14_002, and reference genome (Chase et al. (5)). Only intergenic INDEL regions that ranked among the ten most frequently mutated targets in the present study (Table S1) and were also detected in the prior study are shown. Values for the present study represent the percentage of sequenced isolates harboring at least one mutation at each region, with laboratory isolates pooled across treatments and field isolates pooled across the 12- and 27-month sampling points but reported separately by treatment. Prior study isolate values represent the percentage of recovered isolates containing the mutation, pooled across sampling times and litterbags. Prior study population allele frequencies were estimated from population-level read mapping against the ancestral genome and represent mean allele frequencies across three sampling times (6, 12, and 18 months) from a single litterbag per site. "ND" indicates no detection. Regions were matched across studies using reference genome coordinates.

**Citations**


1. Chase AB, Arevalo P, Polz MF, Berlemont R, Martiny JBH. 2016. Evidence for Ecological Flexibility in the Cosmopolitan Genus Curtobacterium. Front Microbiol 7:1874.
2. Matulich KL, Weihe C, Allison SD, Amend AS, Berlemont R, Goulden ML, Kimball S, Martiny AC, Martiny JBH. 2015. Temporal variation overshadows the response of leaf litter microbial communities to simulated global change. ISME J 9:2477–2489.
3. Chase AB, Karaoz U, Brodie EL, Gomez-Lunar Z, Martiny AC, Martiny JBH. 2017. Microdiversity of an abundant terrestrial bacterium encompasses extensive variation in ecologically relevant traits. mBio 8:e01809-17.
4. Scales NC, Chase AB, Finks SS, Malik AA, Weihe C, Allison SD, Martiny AC, Martiny JBH. 2022. Differential response of bacterial microdiversity to simulated global change. Appl Environ Microbiol 88:e02429-21.
5. Chase AB, Weihe C, Martiny JBH. 2021. Adaptive differentiation and rapid evolution of a soil bacterium along a climate gradient. Proceedings of the National Academy of Sciences 118:e2101254118.
6. Allison SD, Lu Y, Weihe C, Goulden ML, Martiny AC, Treseder KK, Martiny JBH. 2013. Microbial abundance and composition influence litter decomposition response to environmental change. Ecology 94:714–725.
7. Khalili B, Weihe C, Kimball S, Schmidt KT, Martiny JBH. 2019. Optimization of a method to quantify soil bacterial abundance by flow cytometry. mSphere 4:10–1128.
8. Bushnell B. 2014. BBMap: a fast, accurate, splice-aware aligner. Lawrence Berkeley National Lab.(LBNL), Berkeley, CA (United States).
9. Bankevich A, Nurk S, Antipov D, Gurevich AA, Dvorkin M, Kulikov AS, Lesin VM, Nikolenko SI, Pham S, Prjibelski AD. 2012. SPAdes: a new genome assembly algorithm and its applications to single-cell sequencing. Journal of Computational Biology 19:455–477.
10. Chase AB, Gomez-Lunar Z, Lopez AE, Li J, Allison SD, Martiny AC, Martiny JBH. 2018. Emergence of soil bacterial ecotypes along a climate gradient. Environ Microbiol 20:4112–4126.
11. Finks SS, Moudgalya P, Weihe C, Martiny JBH. 2024. The contribution of plasmids to trait diversity in a soil bacterium. ISME communications 4:ycae025.
12. Jain C, Rodriguez-R LM, Phillippy AM, Konstantinidis KT, Aluru S. 2018. High throughput ANI analysis of 90K prokaryotic genomes reveals clear species boundaries. Nat Commun 9.
13. Li H, Durbin R. 2009. Fast and accurate short read alignment with Burrows–Wheeler transform. bioinformatics 25:1754–1760.
14. Li H, Handsaker B, Wysoker A, Fennell T, Ruan J, Homer N, Marth G, Abecasis G, Durbin R, Subgroup 1000 Genome Project Data Processing. 2009. The sequence alignment/map format and SAMtools. bioinformatics 25:2078–2079.
15. Van der Auwera GA, Carneiro MO, Hartl C, Poplin R, Del Angel G, Levy-Moonshine A, Jordan T, Shakir K, Roazen D, Thibault J. 2013. From FastQ data to high-confidence variant calls: the genome analysis toolkit best practices pipeline. Curr Protoc Bioinformatics 43:10–11.
16. Barrick JE, Colburn G, Deatherage DE, Traverse CC, Strand MD, Borges JJ, Knoester DB, Reba A, Meyer AG. 2014. Identifying structural variation in haploid microbial genomes from short-read resequencing data using breseq. BMC Genomics 15:1039.

17. Seemann T. 2014. Prokka: rapid prokaryotic genome annotation. Bioinformatics 30:2068–2069.

18. Kanehisa M, Sato Y, Morishima K. 2016. BlastKOALA and GhostKOALA: KEGG tools for functional characterization of genome and metagenome sequences. J Mol Biol 428:726–731.
